# Exploring the Role of miRNA-101a in the Circulatory System’s Adaptive Mechanisms in Hypertensive Disorders of Pregnancy

**DOI:** 10.3390/diagnostics15050535

**Published:** 2025-02-22

**Authors:** Ewa Szczerba, Eliza Kozyra-Pydyś, Agnieszka Zajkowska, Katarzyna Pankiewicz, Grzegorz Szewczyk, Tomasz Maciejewski, Maciej Małecki, Anna Fijałkowska

**Affiliations:** 1 Department of Cardiology, Institute of Mother and Child, 01-211 Warsaw, Poland; ewa.szczerba@uckwum.pl (E.S.); anna.fijalkowska@imid.med.pl (A.F.); 2 Department of Cardiology, Medical University of Warsaw, 02-097 Warsaw, Poland; 3 Department of Applied Pharmacy, Faculty of Pharmacy, Medical University of Warsaw, 02-097 Warsaw, Poland; agnieszka.zajkowska@wum.edu.pl (A.Z.); maciej.malecki@wum.edu.pl (M.M.); 4 Department of Obstetrics and Gynaecology, Institute of Mother and Child, 01-211 Warsaw, Poland; katarzyna.pankiewicz@imid.med.pl (K.P.); tomasz.maciejewski@imid.med.pl (T.M.); 5 Department of Biophysics, Physiology and Pathophysiology, Medical University of Warsaw, 02-097 Warsaw, Poland; grzegorz.szewczyk@wum.edu.pl; 6 Department of Obstetrics, Gynecology and Gynecological Oncology, Mazovian Voivodship Hospital St John’s Paul 2nd Memorial, 08-110 Siedlce, Poland; 7 Laboratory of Gene Therapy, Faculty of Pharmacy, Medical University of Warsaw, 02-097 Warsaw, Poland

**Keywords:** miRNA-101a, hypertensive disorders of pregnancy, gestational hypertension, NT-proBNP, placental dysfunction

## Abstract

**Background/Objectives:** Hypertensive disorders of pregnancy constitute one of the principal reasons for maternal and perinatal morbidity and mortality worldwide. Identification of molecular mechanisms causing placental dysfunction resulting in gestational hypertension is crucial in the development of new methods of prevention and treatment. The aim of this case-control study was to assess changes in miRNA expression, and biomarkers such as NT-proBNP and galectin-3, in women with uncomplicated pregnancies and hypertensive disorders of pregnancy. **Methods:** This was a case-control study. We included 61 women with uncomplicated pregnancies and 31 women with hypertensive disorders of pregnancy (21 women with gestational hypertension and 10 women with chronic hypertension). Blood sample collection was performed at 33 weeks of gestation. Expression and expression levels of 26 microRNAs, NTproBNP, and galectin-3 were measured. **Results:** Lower expression of microRNA 101a was observed in patients with hypertensive disorders of pregnancy. The expression of microRNA 101a was significantly lower in the group of patients with gestational hypertension, but not with chronic hypertension. Not only was the expression of microRNA 101a lower in all women with gestational hypertension but also in XYZ% it reached undetectable levels. Other studied microRNAs were similar in expression and concentration levels among both groups. In all women with hypertensive disorders of pregnancy, statistically significant correlations were detected between NT-proBNP concentrations and microRNA 133a (r = −0.68; *p* = 0.030) and microRNA 195 (r = 0.67; *p* = 0.030), and between galectin-3 and microRNA 195 (r = 0.46; *p* = 0.010), microRNA 133a (r = 0.44; *p* = 0.020), microRNA 222-2276 (r = 0.39; *p* = 0.050). **Conclusions:** microRNA 101a, a molecule associated with placental dysfunction in preeclampsia and with inhibition of cardiac fibrosis, has lower expression and concentration levels in gestational hypertension but not in chronic hypertension.

## 1. Introduction

Hypertensive disorders complicating pregnancy (HDCP) constitute one of the principal reasons for maternal and perinatal morbidity and mortality worldwide [1,2,3]. Most studies suggest that placental dysfunction is the main cause of gestational hypertension and preeclampsia (PE) and that it leads to pathological changes in the circulatory system [4]. The underlying molecular mechanisms of the changes to cardiac structure and function during pregnancy are not fully understood. Many studies have shown the important role of microRNAs (miRNAs) as potential biomarkers in the prognosis and diagnosis of hypertensive disorders of pregnancy [5]. MicroRNAs are critical mediators in biological processes as they are endogenous, small non-coding RNA molecules that regulate gene expression through post-transcriptive regulation. They influence cellular function profiles and are associated with disease progression [5,6,7].

MicroRNA analyses indicate that a variety of factors cause tissues to display microRNA expression profiles that are significantly different from normal [8]. This may be useful for a wide range of applications in clinical diagnostics [9,10]. MicroRNAs may be responsible for the transient changes in cardiomyocytes and cardiac extracellular matrix cells enabling physiological adaptation to volume overload during pregnancy and adaptation to overload related to hypertension in pregnancy. It was shown that several miRNAs play a role in the pathogenesis of preeclampsia. These include miR-215, miR-155, miR-650, miR-210, and miR-21, which were upregulated, and miR-18a and miR-19b1, which were downregulated in women with PE compared to the control group, and between women with severe and mild PE [11].

Our study aimed to evaluate the differences in miRNA expression between patients with uncomplicated pregnancies and patients with hypertensive disorders of pregnancy in search of factors involved in the pathogenesis of gestational hypertension and adaptation of the cardiovascular system to altered hemodynamic conditions. We evaluated the following miRNAs extracted from the serum of pregnant women: miR-15b, miR-21, miR-26a, miR-26b, and miR-29b were selected, as well as miR-29-a, miR-29c, miR-30c, miR-101a, miR-146a, miR-191, miR-208a, miR-223, and miR-328 as microRNAs associated with myocardial hypertrophy and fibrosis. We also attempted to assess the pathogenetic changes in the circulatory system of a pregnant woman with gestational and chronic hypertension and the correlation between selected miRNAs and biomarkers such as NT-proBNP, galectin-3, and high-sensitivity troponin T.

## 2. Materials and Methods

### 2.1. Study Design

This was a case-control study reported according to STROBE Guidance for observational study [12].

### 2.2. Setting and Participants

The study was conducted between October 2014 and June 2017 at the Institute of Mother and Child, Warsaw, Poland. It included women > 18 years of age with singleton uncomplicated pregnancies or with hypertensive disorders of pregnancy. The protocol was approved by the Local Bioethics Committee, Institute of Mother and Child, Warsaw, Poland (No. 17/2013 from 4 June 2013) and written informed consent from all participants was obtained. Participants underwent a clinical examination, a blood sample collection, and an echocardiographic examination.

### 2.3. Data Sources and Measurement

The samples were taken within 5 days of inclusion in the study, with a median of 33 hbd, an interquartile range for uncomplicated pregnancy of 31–34 hbd, and an interquartile range for hypertensive disorders of pregnancy of 31–35 hbd at the peak volume overload. A detailed description of the experimental procedure, including RNA isolation, cDNA synthesis, and miRNA expression analysis by qPCR, is provided in the authors’ previously published manuscript [13].

### 2.4. Statistical Analysis

miRNA expression is presented as frequencies and as ΔCq values normalized to the U6. Non-parametric tests were used for statistical analysis. Statistical characteristics of continuous variables are presented as medians and ranges or interquartile ranges. Statistical significances of between-group comparisons in the non-parametric variables were verified using a Mann–Whitney U-test. Power analyses were conducted using the effsize and pwr packages in the R programming environment (RStudio 2023.12.0 Build 369, “Ocean Storm” Release, Windows). Initially, effect sizes were calculated for the Mann–Whitney–Wilcoxon tests to quantify the strength of the relationships between the studied variables. Following the determination of effect sizes, power analyses were performed using these values, assuming a significance level (α) of 0.05. The report from the power analysis is provided in Supplementary File S1. Spearman’s rank correlation coefficient analysis was used for the correlation of microRNA with NT-proBNP and galactin-3 levels. All statistical calculations were performed using SAS/STAT version 14.3 (SAS Institute, Inc., Cary, NC, USA). A *p*-value < 0.05 was considered statistically significant.

## 3. Results

### 3.1. Characteristics of Study Groups

We included 61 women with uncomplicated pregnancies and 31 women with hypertensive disorders of pregnancy (21 women with gestational hypertension and 10 women with chronic hypertension). Women with uncomplicated pregnancies and women with hypertensive disorders of pregnancy did not differ in terms of age (31 vs. 32 years, *p* = 0.200) or weeks of gestation (33 vs. 33 HBD, *p* = 0.400). Women with hypertensive disorders of pregnancy had a higher BMI before pregnancy (26.1 vs. 22 kg/m^2^; *p* = 0.005).

### 3.2. Women with Gestational Hypertension Subgroup

The median HBD at the time of gestational hypertension diagnosis was 31 (IQR 24–34). Median maximal measured blood pressure values were 155/97.5 mmHg (IQR for systolic blood pressure 150–160.5 mmHg; IQR for diastolic blood pressure 90–107.5 mmHg). The median blood pressure values were the same for left- and right-arm measurement and equaled 133/85 mmHg. All women were treated with methyldopa, and one additionally with verapamil. Four women had gestational hypertension in previous pregnancies.

### 3.3. Women with Chronic Hypertension Subgroup

The median duration of hypertension before pregnancy was 5 years (IQR 4–7 years). The median of the highest registered blood pressure values was 147.5/94.5 mmHg. Upon physical examination the median blood pressure value for the left arm was 129/77 mmHg and for the right arm was 125/76 mmHg. Seven out of 10 women required pharmacotherapy during pregnancy. Six received methyldopa, one required an additional agent (verapamil), and one was treated with a short-acting metoprolol only. Two women had gestational hypertension in previous pregnancies.

### 3.4. microRNA Expression

**Figure 1:**
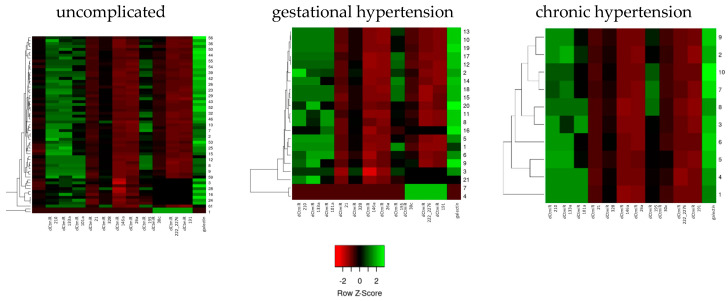
MicroRNA expression profiles in subgroups.

Figure 1 delineates the differential expression patterns of miRNAs across three categorized patient groups. Heatmaps were employed to convey the expression magnitudes, with the Z-score transformed data enabling a standardized view of the expression variations. The gestational hypertension group displayed a noticeable upregulation in specific miRNAs when juxtaposed with the uncomplicated cohort, whereas the chronic hypertension group exhibited a more variegated expression profile. The observed expression patterns underscore the possibility of miRNA involvement in the pathophysiology of hypertensive disorders (Figure 1).

No expression of microRNA 34a or microRNA 208 was found in any of the studied subjects. Expression of microRNA 1 and 199-b was very low and similar between women with uncomplicated pregnancies and with hypertensive disorders of pregnancy. MicroRNA 21, 146-a, 26a, 30c, 222–2276, and 191 was detected in all the subjects. Although statistically insignificant, the expression of miR-124a and mir-21, and profibrotic miRs, was found solely in the gestational hypertension group. The only microRNA with significant differences in expression between subjects with uncomplicated pregnancy and those with hypertensive disorders of pregnancy was microRNA 101a. Its expression was detected in 57% of women with uncomplicated pregnancies and in 35% of women with hypertensive disorders of pregnancy. Further analysis revealed that this difference was a result of a significantly lower expression of microRNA 101a in the gestational hypertension group. In this subgroup, expression of microRNA 101a was found in 24% of women compared to 60% of women with chronic hypertension. Interestingly, this resulted in differences between the uncomplicated pregnancy group and the chronic hypertension group being statistically insignificant (Table 1).

### 3.5. microRNA Concentrations

MicroRNA 101a expression in women with gestational hypertension occurred at lower levels (*p* = 0.009). The concentrations of microRNA 101a in women with uncomplicated pregnancies and chronic hypertension during pregnancy were similar. No other significant differences were detected in concentrations of other studied microRNA (Table 1).

The color coding corresponds to the Z-score row, with green representing lower expression levels and red indicating higher expression levels. The dendrograms on the side of each heatmap represent hierarchical clustering based on expression profiles, highlighting the relative similarities and differences between individual samples.

### 3.6. MicroRNA Correlations with NT-proBNP and Galectin-3 Levels

In all study participants, the NT-proBNP levels were within normal range (<125 pg/mL). Similarly, galectin-3 levels were within the normal reference range, with a threshold of 15.974 ng/mL, as established by Jiang et al. [14].

Interestingly, a mild elevation of high-sensitivity troponin T (hsTnT) levels was observed in the uncomplicated pregnancy group in 17% of cases and in women with hypertensive disorders of pregnancy in 9% of cases. Further analysis showed that this was driven by hsTnT-level elevation in one of the subjects in the chronic hypertension subgroup. None of the women with gestational hypertension had elevated hsTnT levels, which corresponds with the lack of expression of miR-208a-5p, which is also associated with myocardial injury.

In all women with hypertensive disorders of pregnancy, significant correlations were detected between NT-proBNP concentrations and microRNA 133a (r = −0.68; *p* = 0.030) and microRNA 195 (r = 0.67; *p* = 0.030), and also between galectin-3 and microRNA 195 (r = 0.46; *p* = 0.010), microRNA 133a (r = 0.44; *p* = 0.020), and microRNA 222-2276 (r = 0.39; *p* = 0.050).

Several correlations between NT-proBNP and galactin-3 with the studied microRNAs were demonstrated in women with gestational hypertension. NT-proBNP correlated with miR-21, miR-29c, miR 133a, miR-124a, and miR-1249. Galectin-3 concentrations correlated with miR-124a, miR-21, miR-222-2276, miR-133a, miR-191, and miR-1249 (Table 2).

In women with chronic hypertension, concentrations of microRNA 195 correlated with galectin-3 (r = 0.73; *p* = 0.02) only, and none of the studied microRNAs correlated with the concentrations of NT-proBNP. Interestingly, in this subgroup the concentrations of galectin-3 and NT-proBNP demonstrated a strong positive correlation (r = 0.82; *p* = 0.040).

## 4. Discussion

The key findings of this study include significantly lower miR-101a expression in the gestational hypertension group compared to both uncomplicated pregnancies and chronic hypertension cases. NT-proBNP and galectin-3 levels remained within normal ranges for all participants. Significant correlations were observed between certain miRNAs (e.g., miR-133a, miR-195) and NT-proBNP or galectin-3 levels, particularly in the gestational hypertension subgroup. None of the women with gestational hypertension had elevated hsTnT levels, which corresponds with the lack of expression of miR-208a-5p, which is also associated with myocardial injury.

The majority of existing studies on the role of microRNA in pregnancy and pregnancy complicated by gestational hypertension focus on placental microRNA [11]. Notably, microRNA was linked with cellular differentiation, immunomodulation, embryogenesis, and pregnancy complications such as preeclampsia, intrauterine growth restriction, or preterm labor. In the case of preeclampsia, the most extensively studied miRNAs are miR-155 and miR-210 [13,14,15,16,17,18,19,20,21].

Building upon the foundational understanding of placental microRNA dynamics, recent studies have begun exploring the role of circulating microRNAs in maternal serum as potential biomarkers for hypertensive disorders complicating pregnancy (HDCPs). These studies underscore the significance of microRNAs in the placenta and systemic circulation, offering new avenues for early detection and management of these disorders. In particular, miR-19a, miR-126, and miR-210 demonstrate distinct expression patterns between normal pregnancies and those affected by HDCPs, with correlations to disease severity. These findings suggest their utility as sensitive biomarkers for early diagnosis and monitoring of HDCP progression [20,22].

Furthermore, microRNAs such as miR-200a-3p and miR-204 have garnered attention for their diagnostic and prognostic capabilities within the HDCP spectrum. miR-200a-3p has been particularly noted for its ability to predict adverse pregnancy outcomes and is strongly correlated with clinical indices that are indicative of the severity of the disorder. This microRNA’s elevated expression levels in patients with HDCPs suggest its potential role in diagnosing the condition and predicting its progression, thus offering a strategic target for monitoring and potentially mitigating adverse outcomes [21,23]. Additionally, miR-204’s diagnostic value is underscored by its association with inflammatory markers and its demonstrated capacity to predict adverse pregnancy outcomes, making it a useful marker for assessing the risk of complications in HDCPs. The robust predictive power of miR-204 in HDCPs suggests that elevated levels could serve as an early warning sign, potentially guiding interventions to prevent severe manifestations of the disorder [21,22,23,24].

Our focus on microRNAs associated with cardiac adaptation provides a new insight on the subject of molecular changes in hypertensive disorders of pregnancy, emphasizing the role of improper circulatory system adaptation as a mechanism for the development of complications and a target for treatment. We had shown that there is no evidence of a greater cardiomyocyte injury in hypertensive disorders of pregnancy compared to uncomplicated pregnancies—the percentage of patients with elevated troponin levels was similar between both groups, and expression of miR-208 was not found in any of the patients. No significant changes were observed in expression or in concentrations of microRNAs previously associated with hypertrophy, heart failure, and ischemic heart diseases. In concordance with these observations, no significant differences were observed in NT-proBNP concentrations—a commonly used parameter of cardiac overload, and in galectin-3 levels—a well-recognized marker of cardiac fibrosis. It is noteworthy that in our study, no increase in miR-29a was observed, which contrasts with the findings reported by Khaliq et al. They demonstrated that serum levels of miR-29a were elevated in both preeclampsia and gestational hypertension patients compared to normotensive patients, highlighting the distinct yet related molecular profiles between these disorders [23,25].

Similarly to the observed discrepancy in miR-29a levels, our study also found no increase in miR-210, which deviates from the findings reported by Jin et al. [20,22]. In their study, miR-210 was shown, along with miR-19a and miR-126, to exhibit distinct expression patterns in pregnancies affected by hypertensive disorders complicating pregnancy, correlating with disease severity [22]. Notably, the study by Toljic et al. did not observe significant changes in the expression levels of miR-29a, miR-17, or miR-181a in gestational hypertension, despite detecting an upregulation of TNF-α, which correlated positively with IL-1β and IL-17 but negatively with miR-181a. These findings suggest that while inflammatory cytokines play a role in gestational hypertension, the expected alterations in specific microRNAs, including miR-210, may not be universally present across different cohorts or methodological settings [26].

Additionally, Hromadnikova et al. investigated the expression of microRNAs associated with cardiovascular and cerebrovascular diseases but assessed this in full umbilical cord blood, comparing gestational hypertension patients and uncomplicated pregnancy. Their findings indicated a down-regulation of miR-195-5p in HDCP [17,19]. Our group has previously reported that microRNA 101a is upregulated in uncomplicated pregnancies compared to nonpregnant women [24,27]. In this study, we demonstrated that the expression of microRNA 101a in women with gestational hypertension occurred at significantly lower levels compared to women with uncomplicated pregnancies and those with chronic hypertension during pregnancy. The similarity in microRNA 101a concentrations between the latter two groups suggests that this microRNA may be specifically associated with gestational hypertension.

On one hand, the observed decreased expression of miR-101a in our study could be associated with cardiac adaptation to pregnancy. Existing research, including findings by Dong et al., suggests a connection between down-regulated miR-101 and rheumatic heart disease, indicating a broader role of this microRNA in cardiovascular conditions [25,28]. Additionally, various studies have highlighted the regulatory effects of miR-101a on fibrosis. For instance, upregulation of miR-101a has been shown to suppress chronic renal fibrosis by targeting KDM3A [22,24], and it has been linked with anti-fibrotic capacities through the inhibition of TGF-beta 1 receptor. In animal models, cardiac fibrosis is mitigated by the upregulation of miR-101a [26,27,29,30]. There are also reports suggesting a proangiogenic role for microRNA 101a in myocardial infarction models in animals [28,31].

On the other hand, a study conducted in 2009 by Zhu et al. on placental tissue revealed differences in the expression of miR-101a between groups with and without preeclampsia [29,32]. Additionally, research has shown that miR-101 is inversely correlated with ERp44 in preeclampsia placentas, and its upregulation in vitro can inhibit apoptosis in trophoblast cells by targeting ERp44, suggesting a protective role against ER stress-induced apoptosis [30,33].

Furthermore, an experimental study was conducted to evaluate the role of extracellular vesicle (EV)-encapsulated microRNA miR-101 in the biological processes of trophoblasts in preeclampsia and its underlying mechanisms. EV-encapsulated miR-101, derived from human umbilical cord mesenchymal stem cells, was demonstrated to enhance the proliferation and migration of trophoblast cells by targeting and negatively regulating the expression of bromodomain-containing protein 4. This interaction led to the suppression of the NF-κB/CXCL11 signaling pathway, which is crucial for cell behavior. Further in vivo evidence showed that this treatment not only reduced blood pressure but also decreased urine protein levels in a rat model of PE, offering promising therapeutic potential for managing this condition [31,34].

Our findings align with those reported in the study by Li et al., highlighting the potential of miR-101a as a diagnostic marker. Their research demonstrated that serum miR-101 levels were down-regulated and inversely correlated with soluble fms-like tyrosine kinase-1 (sFlt-1), establishing miR-101 as an independent risk factor for hypertensive disorder complicating pregnancy and revealing its decrease with increasing severity of HDCPs. The diagnostic precision of miR-101, indicated by the area under the curve, was substantial in differentiating between stages of the disorder, such as from gestational hypertension to mild preeclampsia, and from mild preeclampsia to severe preeclampsia. These correlations further validate the utility of miR-101a as a reliable biomarker for assessing and grading HDCPs, thereby enhancing its potential for targeted diagnostic applications [16,18].

Given the considerations above, the decreased expression of miR-101a in pregnancies complicated by hypertension is unequivocal. Nonetheless, the precise mechanism remains unknown and warrants further investigation. Our study suggests the importance of interplay between placenta and cardiac adaptation in hypertensive disorders of pregnancy. Improper placenta formation along with the inability of the maternal circulatory system to adapt to new hemodynamic conditions lead to the development of gestational hypertension and in some cases to preeclampsia, eclampsia, or peripartum cardiomyopathy and can result in serious and even deadly complications.

Although no significant differences in NT-proBNP, galectin-3 levels, or miRNA expression (miR-21, miR-29c, miR-133a, miR-124a, miR-1249, miR-222-2276) were observed across the study groups, we identified significant correlations with varying strengths between these biomarkers and miRNAs. To the best of our knowledge, such correlations have not been previously described in the literature. While the associations between NT-proBNP, galectin-3, and cardiac remodeling, particularly under pathological conditions such as heart failure and hypertensive disorders, are well documented, further studies are necessary to elucidate the mechanisms underlying these newly discovered correlations. In our study, none of the women with gestational hypertension had elevated high-sensitivity troponin T levels. This is in contrast to the results of Ravichandran et al., who assessed the concentration of high-sensitivity troponin I (hs-TnI) in patients with physiological pregnancies and pregnancies complicated by hypertension. In their study, patients with pregnancy-induced hypertension and pre-eclampsia had higher concentrations of hs-TnI. In our study, women with gestational hypertension had normal levels of hsTnT, which corresponds to the lack of expression of miR-208a-5p, which is associated with myocardial injury. Understanding these interactions could provide further insights into the pathophysiology of hypertensive disorders of pregnancy and potentially unveil new biomarkers or therapeutic targets.

Medications taken during pregnancy may play a significant role in influencing the levels of the studied biomarkers, such as NT-proBNP, galectin-3, and miR-10. Beta-agonists, which are often used to manage preterm labor, can exacerbate cardiac load and potentially increase NT-proBNP levels. For instance, ritodrine, a beta-agonist, has been shown to cause tachycardia and increased cardiac output, which may lead to elevated NT-proBNP levels due to the increased strain on the heart [35]. Furthermore, certain antihypertensive medications, such as calcium channel blockers, may also influence NT-proBNP levels by modulating vascular resistance and cardiac workload [36]. Non-steroidal anti-inflammatory drugs (NSAIDs), commonly used for pain management, can alter galectin-3 levels by affecting inflammatory pathways [37]. Additionally, medications that modulate the immune response, such as corticosteroids, may also impact galectin-3 expression, potentially influencing placental function and maternal immune tolerance [38]. Research indicates that certain medications, such as those affecting the immune system or hormonal balance, can alter the expression of miR-101a. For example, glucocorticoids, which are often prescribed for autoimmune conditions during pregnancy, have been shown to modulate microRNA expression profiles, including miR-101a, potentially impacting inflammatory responses and tissue remodeling [39]. Furthermore, the use of antiepileptic drugs during pregnancy has been associated with changes in microRNA expression, which could affect fetal development and maternal health [40]. Understanding the interactions between medications and these biomarkers is important for optimizing diagnosis and treatment strategies, and improving outcomes for mothers and infants. To the best of our best knowledge, no information is available in the literature regarding the effect of methyldopa, a drug used in pregnancy for PIH treatment, on the examined markers.

### Limitations

The main limitation of our research is the size of our study groups, particularly within the sub-analysis of different types of hypertensive disorders of pregnancy. Consequently, the results are preliminary and necessitate further investigation with larger cohorts. To mitigate the impact of the limited sample sizes, we employed robust statistical analyses, including power analysis. This power analysis indicates that the small number of observations may result in undetected relationships beyond those we identified. Nevertheless, this limitation does not undermine the validity of the positive findings we observed, which warrant consideration and further exploration in future studies. Another limitation is the influence of some of the patients’ characteristics on the observed results. The influence of antihypertensive treatment and BMI warrants special emphasis. All women from the gestational hypertension subgroup and most of the women (70%) with chronic hypertension were treated with methyldopa, with only a single patient treated with verapamil. This does not allow for the analysis of the influence of the antihypertensive drugs on the expression of microRNA in our study population, and requires further study. There were significant differences in BMI values between women with uncomplicated pregnancies and hypertensive disorders of pregnancy, caused by a higher body mass index of women with gestational hypertension. This can be explained by excess weight and obesity being known risk factors for gestational hypertension; however, this could have also impacted the observed results.

## 5. Conclusions

In women with gestational hypertension, there is a lower expression and lower concentration of microRNA 101a compared to women with uncomplicated pregnancies and pregnant women with chronic hypertension. Expression levels and concentrations of other microRNAs associated with cardiac hypertrophy and fibrosis are similar in uncomplicated pregnancy and both types of studied hypertensive disorders of pregnancy. Our results suggest a role of miR 101a in circulatory system adaptation in hypertensive disorders of pregnancy. These findings indicate the possibility of using mir101a expression levels in maternal blood for the diagnosis of hypertensive disorders of pregnancy.

## Figures and Tables

**Table 1:** Characteristics and cardiovascular biomarkers and miRNA expression among groups.

Parameter	UNP (*N* = 61) M [IQR] *N* (%)	HDP (*N* = 31) M [IQR] *N* (%)	GH (*N* = 21) M [IQR] *N* (%)	ChH (*N* = 10) M [IQR] *N* (%)	UNP vs. HDP *p*-Value	UNP vs. GH *p*-Value	UNP vs. ChH *p*-Value
NT-proBNP	37.1 [22–52.2]	67.6 [27.2–100.1]	75.1 [35.4–99.5]	44.7 [27.2–108.1]	0.103	0.137	0.306
TnT > *N*	5 (17%)	1 (9%)	0	1/6	0.543	0.324	1.000
Galectin-3	10.6 [8.2–13.6]	11.8 [9.2–13.2]	12 [8.8–13.1]	10.9 [9.8–13.2]	0.473	0.620	0.513
age	31 [27–35]	32 [30–35]	32 [29–34]	34.5 [31–36]	0.218	0.652	0.087
HBD in enrolment	33 [31–34]	33 [31–35]	35 [33–36]	31.5 [30–33]	0.392	0.037	0.120
BMI before preg	22 [20.0–24.7]	26.1 [22.6–27.9]	26 [22.9–29.3]	26.3 [20.4–27.7]	0.005	0.008	0.191
miR 210 ex	22 (36%)	8 (26%)	6 (29%)	2 (20%)	0.321	0.532	0.320
miR 133a ex	17 (28%)	6 (19%)	5 (24%)	1 (10%)	0.373	0.717	0.229
**miR 101-a ex**	**35 (57%)**	**11 (35%)**	**5 (24%)**	6 (60%)	**0.047**	**0.008**	0.876
miR 15b ex	58 (95%)	29 (94%)	19 (90%)	10 (100%)	0.759	0.447	0.474
miR-27b ex	41 (67%)	18 (58%)	11 (52%)	7 (70%)	0.387	0.224	0.861
miR-21 ex	61 (100%)	31 (100%)	21 (100%)	10 (100%)	1.000	1.000	1.000
miR-1 ex	1 (2%)	2 (6%)	1 (5%)	1 (10%)	0.219	0.424	0.139
miR-328 ex	58 (95%)	29 (94%)	19 (90%)	10 (100%)	0.759	0.447	0.474
miR-34a ex	0	0	0	0	N/A	N/A	N/A
miR-222 ex	58 (95%)	31 (100%)	21 (100%)	10 (100%)	0.209	0.301	0.474
miR-208a-5p ex	0	0	0	0	N/A	N/A	N/A
miR-146a ex	61 (100%)	31 (100%)	21 (100%)	10 (100%)	1.000	1.000	1.000
miR-199b ex	2 (3%)	0	0	0	0.308	0.401	0.561
miR-199a-3p ex	60 (98%)	30 (97%)	20 (95%)	10 (100%)	0.622	0.424	0.684
miR-29c ex	60 (98%)	29 (94%)	19 (90%)	10 (100%)	0.219	0.097	0.683
miR-26a ex	61 (100%)	31 (100%)	21 (100%)	10 (100%)	1.000	1.000	1.000
miR-29a ex	57 (93%)	29 (94%)	20 (95%)	9 (90%)	0.985	0.767	0.693
miR-195 ex	40 (66%)	21 (68%)	14 (67%)	7 (70%)	0.835	0.927	0.784
miR-17-5p ex	58 (95%)	31 (100%)	21 (100%)	10 (100%)	0.209	0.301	0.474
miR-30c ex	61 (100%)	31 (100%)	21 (100%)	10 (100%)	1.000	1.000	1.000
miR-22 ex	33 (54%)	17 (55%)	13 (62%)	4/10	0.946	0.534	0.408
miR-222-2276 ex	61 (100%)	31 (100%)	21 (100%)	10 (100%)	1.000	1.000	1.000
miR-191 ex	61 (100%)	31 (100%)	21 (100%)	10 (100%)	1.000	1.000	1.000
miR-1249 ex	17 (28%)	7 (23%)	6 (29%)	1 (10%)	0.585	0.951	0.229
miR-124a ex	11 (18%)	5 (16%)	5 (24%)	0	0.820	0.565	0.144
dCT miR-210	7.7 [5.9–8.9]	8 [6.4–9.5]	7.8 [6.3–9.5]	8.0 [7.9–9.7]	0.169	0.453	0.115
dCT miR-133a	7.95 [5.1–9.3]	8.3 [6.6–9.7]	8.3 [5.6–9.5]	8.7 [7.6–9.7]	0.222	0.459	0.199
**dCT miR-101a**	**4.6 [2.7–7.7]**	7.7 [3.4–9.4]	**7.7 [5.6–9.4]**	4.3 [1.6–8.0]	0.054	**0.009**	0.811
dCT miR-15b	1.9 [1.1–2.7]	2.5 [0.5–3.9]	2.7 [1–4]	0.7 [−0.5–2.7]	0.593	0.120	0.177
dCT miR-27b	3.1 [2–6.4]	3.7 [2–8.3]	3.7 [2.4–8.3]	3.6 [1.5–8.0]	0.418	0.334	0.915
dCT miR-21	−1 [−1.9–0.4]	−1.2 [−1.8–0.8]	−1.2 [−1.6–0.1]	−1.3 [−2.0–0.3]	0.805	0.975	0.582
dCT miR-1	8.9 [7.9–9.8]	9.1 [7.7–9.6]	9.1 [7.7–9.7]	8.7 [7.9–9.7]	0.761	0.695	1.000
dCT miR-328	1.6 [0.9–3.2]	2.3 [0.8–3.0]	2.4 [1.5–3.2]	0.8 [0.3–2.3]	0.705	0.133	0.115
dCT miR-222	−0.2 [−1.2–1.6]	−0.2 [−1.5–1.6]	0.4 [−1.7–1.3]	−0.6 [−1.3–1.9]	0.938	0.861	0.663
dCT miR-146a	−2.5 [−3.4–1.8]	−2.4 [−3.7–1.6]	−2 [−3.4–1.6]	−3.2 [−3.8—1.9]	0.866	0.485	0.460
dCT miR-199b	8.9 [7.9–9.6]	9.2 [7.7–9.7]	9.2 [7.7–9.6]	8.7 [7.9–9.7]	0.773	0.816	0.837
dCT miR-29c	0.5 [−0.4–2.7]	0.4 [−0.5–2.4]	1.1 [0.1–3.5]	−0.4 [−0.8–1.3]	0.984	0.395	0.172
dCT miR-26a	−2.2 [−3.1–1.2]	−1.8 [−3.5–1.2]	−1.7 [−2.5–1.2]	−3.5 [−5.3–1.2]	0.994	0.375	0.162
dCT miR-199a-3p	0.74 [0.04–1.7]	1 [−0.6–2.2]	1 [0.2–2.1]	0.2 [−0.6–2.2]	0.850	0.597	0.663
dCT miR-29a	1.5 [0.4–2.4]	1.6 [0.8–2.8]	1.6 [0.8–2.7]	1.8 [0.5–3.7]	0.320	0.404	0.495
dCT miR-195	3.8 [2.2–7.2]	3.7 [1.7–7.2]	3.7 [2.2–7.2]	2.5 [1.7–6.8]	0.902	0.849	0.582
dCT miR-17-5p	1.7 [0.7–2.8]	1.5 [0.6–3]	1.6 [0.9–2.6]	1.2 [0.4–3.4]	0.707	0.958	0.546
dCT miR-30c	0.2 [−0.7–1.3]	0.9 [−1.1–1.3]	1 [−0.4–1.6]	−0.7 [−1.3–1.2]	0.575	0.180	0.448
dCT miR-22	6.3 [4.7–8.4]	7.5 [4.5–9.4]	7.1 [4.9–9.5]	7.9 [4–9.3]	0.582	0.527	0.882
dCT miR-222-2276	−2.5 [−3.1–1.8]	−2.5 [−3–1.5]	−2 [−2.5–1.5]	−2.8 [−3.2–1.5]	0.510	0.195	0.601
dCT miR-191	−2.3 [−3.3–1.5]	−2.1 [−3.5–1.3]	−1.9 [−3.1–1.2]	−3.0 [−3.7–1.3]	0.857	0.309	0.311
dCT miR-1249	8.5 [7.7–9.3]	9.2 [7.8–9.7]	9.4 [8.1–9.7]	8.0 [7.6–9.4]	0.167	0.074	0.914
dCT miR-124a	8.8 [7.7–9.6]	9.4 [8–9.7]	9.5 [8.7–9.7]	8.7 [7.9–9.7]	0.222	0.149	0.775

UNP—uncomplicated pregnancy; HDP—any hypertension disorder; GH—gestational hypertension; ChH—chronic hypertension; bold font was used to demonstrate statistically significant values.

**Table 2:** Significant correlation between certain microRNAs and NT-proBNP and galectin-3 in the gestational hypertension group.

miR	Correlation with NT-proBNP	Correlation with Galectin-3
miR-21	r = −1.0; *p* < 0.001	r = 0.592; *p* = 0.016
miR-29c	r = −1.0; *p* < 0.001	r = 0.368; *p* = 0.161
miR-133a	r = −1.0; *p* < 0.001	r = 0.567; *p* = 0.009
miR-124a	r = −1.0; *p* < 0.001	r = 0.592; *p* = 0.016
miR-1249	r = −1.0; *p* < 0.001	r = 0.536; *p* = 0.032
miR-222-2276	r = 0.8; *p* = 0.200	r = 0.582; *p* = 0.018

## Data Availability

Data are available upon request from the corresponding author.

## Supplementary Materials

The following supporting information can be downloaded at: https://www.mdpi.com/article/10.3390/diagnostics15050535/s1, File S1: Analysis of statistical test power and effect size.
