# Supplementary material for: Exploring the Role of miRNA-101a in the Circulatory System’s Adaptive Mechanisms in Hypertensive Disorders of Pregnancy
Source: Diagnostics (Basel). 2025 Feb 22;15(5):535. doi: 10.3390/diagnostics15050535 (PMC11899601; doi:10.3390/diagnostics15050535)
Supplement: Supplementary file 1 [file diagnostics-15-00535-s001.zip › diagnostics-3396051-supplementary.pdf]

ANALYSIS OF STATISTICAL TEST POWER AND EFFECT SIZE

The power of the test is shown in the power column. It is given in the range of 0 to 1, expressing the probability of accepting the null hypothesis when it is false. The closer it is to 1, the greater the chance that the test result shows a true, significant difference between the parameters. Effsize is the effect size - the strength of the relationship between the variables under study. We consider that at a level up to 0.3 it is weak, around 0.5 it is average, and above 0.7 it is strong. This value can exceed 1 for very strong relationships.

variable	group1	group2	effsize	magnitude	N	power
dCt miR-195	UNP	HDP	0.013349	small	92	0.05093
dCt miR-195	UNP	GH	0.021704	small	82	0.052189
dCt miR-195	UNP	ChH	0.066697	small	71	0.068028
dCt miR-195	GH	ChH	0.075897	small	31	0.059963
dCt miR-210	UNP	HDP	0.145114	small	92	0.164958
dCt miR-210	UNP	GH	0.083883	small	82	0.083248
dCt miR-210	UNP	ChH	0.190284	small	71	0.203159
dCt miR-210	GH	ChH	0.189743	small	31	0.113835
dCt miR-29a	UNP	HDP	0.104637	small	92	0.108757
dCt miR-29a	UNP	GH	0.093269	small	82	0.091255
dCt miR-29a	UNP	ChH	0.082391	small	71	0.077646
dCt miR-29a	GH	ChH	0.045538	small	31	0.053573
dCtmiR-1	UNP	HDP	0.032295	small	92	0.055456
dCtmiR-1	UNP	GH	0.043995	small	82	0.059031
dCtmiR-1	UNP	ChH	0	small	71	0.05
dCtmiR-1	GH	ChH	0.075897	small	31	0.059963
dCtmiR-101a	UNP	HDP	0.203676	small	92	0.279416
dCtmiR-101a	UNP	GH	0.295058	small	82	0.467436
dCtmiR-101a	UNP	ChH	0.029425	small	71	0.05348
dCtmiR-101a	GH	ChH	0.227691	small	31	0.142795
dCtmiR-1249	UNP	HDP	0.16076	small	76	0.16632
dCtmiR-1249	UNP	GH	0.224696	small	66	0.249257
dCtmiR-1249	UNP	ChH	0.015364	small	60	0.050799
dCtmiR-1249	GH	ChH	0.206725	small	26	0.113127
dCtmiR-124a	UNP	HDP	0.141921	small	76	0.140036
dCtmiR-124a	UNP	GH	0.180494	small	66	0.177389
dCtmiR-124a	UNP	ChH	0.038411	small	60	0.055003
dCtmiR-124a	GH	ChH	0.134371	small	26	0.07625
dCtmiR-133a	UNP	HDP	0.128751	small	92	0.139901
dCtmiR-133a	UNP	GH	0.08271	small	82	0.08231
dCtmiR-133a	UNP	ChH	0.154973	small	71	0.150499
dCtmiR-133a	GH	ChH	0.136615	small	31	0.082663
dCtmiR-146a	UNP	HDP	0.018085	small	92	0.051707

dCtmiR-146a	UNP	GH	0.078017	small	82	0.078698
dCtmiR-146a	UNP	ChH	0.089258	small	71	0.082523
dCtmiR-146a	GH	ChH	0.182153	small	31	0.108717
dCtmiR15b	UNP	HDP	0.056409	small	92	0.066751
dCtmiR15b	UNP	GH	0.174219	small	82	0.198453
dCtmiR15b	UNP	ChH	0.16282	small	71	0.161236
dCtmiR15b	GH	ChH	0.387075	moderate	31	0.32288
dCtmiR-17-5p	UNP	HDP	0.043958	small	76	0.058345
dCtmiR-17-5p	UNP	GH	0.007367	small	66	0.050202
dCtmiR-17-5p	UNP	ChH	0.079383	small	60	0.071566
dCtmiR-17-5p	GH	ChH	0.093026	small	26	0.062491
dCtmiR-191	UNP	HDP	0.021351	small	76	0.051961
dCtmiR-191	UNP	GH	0.127082	small	66	0.111988
dCtmiR-191	UNP	ChH	0.133158	small	60	0.111772
dCtmiR-191	GH	ChH	0.248069	small	26	0.141761
dCtmiR-199a-3p	UNP	HDP	0.020238	small	92	0.052138
dCtmiR-199a-3p	UNP	GH	0.059246	small	82	0.066446
dCtmiR-199a-3p	UNP	ChH	0.052966	small	71	0.061327
dCtmiR-199a-3p	GH	ChH	0.136615	small	31	0.082663
dCtmiR-199b	UNP	HDP	0.030573	small	92	0.054888
dCtmiR-199b	UNP	GH	0.026397	small	82	0.05324
dCtmiR-199b	UNP	ChH	0.025502	small	71	0.052613
dCtmiR-199b	GH	ChH	0.022769	small	31	0.050892
dCtmiR-21	UNP	HDP	0.027128	small	92	0.053846
dCtmiR-21	UNP	GH	0.002933	small	82	0.05004
dCtmiR-21	UNP	ChH	0.066697	small	71	0.068028
dCtmiR-21	GH	ChH	0.113846	small	31	0.07257
dCtmiR-22	UNP	HDP	0.064053	small	76	0.067812
dCtmiR-22	UNP	GH	0.079196	small	66	0.073672
dCtmiR-22	UNP	ChH	0.020486	small	60	0.05142
dCtmiR-22	GH	ChH	0.031009	small	26	0.051379
dCtmIR-222	UNP	HDP	0.008612	small	92	0.050387
dCtmIR-222	UNP	GH	0.019944	small	82	0.051848
dCtmIR-222	UNP	ChH	0.052966	small	71	0.061327
dCtmIR-222	GH	ChH	0.022769	small	31	0.050892
dCtmiR-222-2276	UNP	HDP	0.076612	small	76	0.075584
dCtmiR-222-2276	UNP	GH	0.162076	small	66	0.152116
dCtmiR-222-2276	UNP	ChH	0.06914	small	60	0.066314
dCtmiR-222-2276	GH	ChH	0.258406	small	26	0.14979
dCtmiR-26a	UNP	HDP	0.001292	small	92	0.050009
dCtmiR-26a	UNP	GH	0.099135	small	82	0.096718
dCtmiR-26a	UNP	ChH	0.168705	small	71	0.169654
dCtmiR-26a	GH	ChH	0.288409	small	31	0.200766
dCtmiR-27b	UNP	HDP	0.08526	small	92	0.088684
dCtmiR-27b	UNP	GH	0.107934	small	82	0.105581

dCtmiR-27b	UNP	ChH	0.013732	small	71	0.050757
dCtmiR-27b	GH	ChH	0.075897	small	31	0.059963
dCtmiR-29c	UNP	HDP	0.003445	small	92	0.050062
dCtmiR-29c	UNP	GH	0.093856	small	82	0.091786
dCtmiR-29c	UNP	ChH	0.164782	small	71	0.164007
dCtmiR-29c	GH	ChH	0.417434	moderate	31	0.366041
dCtmiR-30c	UNP	HDP	0.065309	small	76	0.068524
dCtmiR-30c	UNP	GH	0.167601	small	66	0.1594
dCtmiR-30c	UNP	ChH	0.099869	small	60	0.084349
dCtmiR-30c	GH	ChH	0.217061	small	26	0.119764
dCtmiR-328	UNP	HDP	0.040046	small	92	0.058403
dCtmiR-328	UNP	GH	0.168353	small	82	0.18839
dCtmiR-328	UNP	ChH	0.190284	small	71	0.203159
dCtmiR-328	GH	ChH	0.417434	moderate	31	0.366041
Galectin-3	UNP	HDP	0.077429	small	88	0.080384
Galectin-3	UNP	GH	0.057022	small	78	0.064467
Galectin-3	UNP	ChH	0.08086	small	68	0.075459
Galectin-3	GH	ChH	0.024102	small	30	0.050966
NTproBNP pg/ml	UNP	HDP	0.183813	small	41	0.130328
NTproBNP pg/ml	UNP	GH	0.119523	small	35	0.078274
NTproBNP pg/ml	UNP	ChH	0.176865	small	36	0.114729
NTproBNP pg/ml	GH	ChH	0.220193	small	11	0.078165
TnT ng/ml	UNP	HDP	0.074738	small	41	0.062902
TnT ng/ml	UNP	GH	0.163646	small	35	0.103603
TnT ng/ml	UNP	ChH	0.027243	small	36	0.05149
TnT ng/ml	GH	ChH	0.275241	small	11	0.094332

UNP – uncomplicated pregnancy; HDP – any hypertension disorder; GH – gestational hypertension; ChH – chronic hypertension.
